# The Greenhouse Gas Emission from Portland Cement Concrete Pavement Construction in China

**DOI:** 10.3390/ijerph13070632

**Published:** 2016-06-24

**Authors:** Feng Ma, Aimin Sha, Panpan Yang, Yue Huang

**Affiliations:** 1 Key Laboratory Special Area Highway Engineering of Ministry of Education , Chang’an University, Xi’an 710064, China; mafeng@chd.edu.cn (F.M.); ppyangchd@sina.com (P.Y.); 2 School of the Civil Engineering, Liverpool John Moores University, Peter Jost Enterprise Centre, Byrom Street, Liverpool L3 3AF, UK; Y.Huang@ljmu.ac.uk

**Keywords:** highway engineering, greenhouse gas (GHG), Portland cement concrete pavement, construction process

## Abstract

This study proposes an inventory analysis method to evaluate the greenhouse gas (GHG) emissions from Portland cement concrete pavement construction, based on a case project in the west of China. The concrete pavement construction process was divided into three phases, namely raw material production, concrete manufacture and pavement onsite construction. The GHG emissions of the three phases are analyzed by a life cycle inventory method. The CO_2_e is used to indicate the GHG emissions. The results show that for 1 km Portland cement concrete pavement construction, the total CO_2_e is 8215.31 tons. Based on the evaluation results, the CO_2_e of the raw material production phase is 7617.27 tons, accounting for 92.7% of the total GHG emissions; the CO_2_e of the concrete manufacture phase is 598,033.10 kg, accounting for 7.2% of the total GHG emissions. Lastly, the CO_2_e of the pavement onsite construction phase is 8396.59 kg, accounting for only 0.1% of the total GHG emissions. The main greenhouse gas is CO_2_ in each phase, which accounts for more than 98% of total emissions. N_2_O and CH_4_ emissions are relatively insignificant.

## 1. Introduction

Environmental issues are becoming an increasing priority for both the government and the private sector. The emphasis has gradually shifted from a site-specific focus on environmental degradation to include the product supply chain. Greenhouse gases (GHG) and their effect on the climate have been in the spotlight with respect to policy and legislation, as well as to general concern by the public. Perceived as an invaluable asset for the development of a robust economy, the highway network has become a primary mode of transportation and a driver of economic growth in China. Large investments were put into the highway infrastructure. Consideration of the environmental impacts of building such a system, however, only began recently. Green construction and sustainable development have emerged as a solution to the conflict between our growing economy and the weakened environment. In addition to traditional technical objectives, environmental impact and sustainability are increasingly being considered in the construction of highways in China.

In this study, an inventory analysis is derived from Life Cycle Assessment (LCA). LCA includes life cycle inventory analysis and life cycle environmental impact analysis. The former includes developing the standard of data acquirement, organizing industrial inventory content, developing statistical models, processing data through a statistical approach and an input-output approach. Life cycle impact assessments include the study of the indicator system, characterization of inventory results, and normalizing the format of the inventory report. The raw materials of concrete pavement projects include aggregates (mainly sand and gravel), cement, steel (for reinforced concrete), additive agents and water.

## 2. Literature Review

Zapata et al. presented a study to assess asphalt pavement and continuously reinforced concrete pavement in terms of energy consumption. The research suggested that for the same service life, cement concrete pavements require more energy in the extraction of raw materials, manufacturing and placing of pavement materials. Additionally, most energy is consumed in the manufacturing process of cement and reinforcing steel. For asphalt pavement, the major consumption of energy is from mixing, the drying of aggregate and the production of bitumen [1].

Stripple et al. conducted a life cycle inventory study of road projects with different geotechnical and meteorological conditions in Sweden to calculate the energy consumption of highway construction, maintenance, and operation [2]. The sum values are shown for the initial construction, the maintenance, and the operation activities as well as the total sum of the entire system.

The Athena Institute presented an assessment of energy consumption and environment burden for the construction and maintenance of comparable asphalt pavement and cement concrete pavement structures in Canada. For the arterial and high-volume highways, neither material design has a distinct advantage in terms of global warming potential (GWP) effects. These differences range from less than 1% to as much as 7% [3].

Huang et al. examined methodological choices made by a UK specification for the assessment of GHG emissions from pavement. They developed an LCA model for pavement construction and maintenance that accommodates recycled materials, such as waste glass, incinerator bottom ash, and recycled asphalt pavement. The results suggested that the production of hot mix asphalt and bitumen was an energy-intensive process [4,5].

Along with rapid urbanization, Philip White et al. presented a process to model the climate change impacts of highway material production, as well as the construction of asphalt and cement concrete pavement. The process presented employs variables that can be modified by the designer to customize for their specific highway pavement design and type of materials [6].

According to Choate’s research, cement manufacturing and concrete production are bound together in the Life Cycle Analysis of energy use and emissions. Concrete accounts for about 20% of the energy and 12% of the CO_2_ emissions associated with cement/concrete in the USA. More than 104 million tons of CO_2_ emissions were associated with US quarrying, cement manufacturing and concrete production [7].

Horvath et al. conducted a study of a Life Cycle Inventory Analysis for asphalt and steel-reinforced concrete pavements. The research results showed that the asphalt pavements may be an environmentally better choice if the asphalt pavements are recycled effectively. Based on the uncertainty of the data and the environmental effects, the resource input requirements and environmental impacts of asphalt and reinforced concrete pavements appear to be roughly comparable [8]. 

The GHG emissions and environmental burden related to highways and vehicles have attracted the interest of researchers for the last 20 years [9,10,11,12,13]. The carbon footprint of asphalt pavement is evaluated in different countries. In the highway construction process, the materials extraction and production, transportation, and onsite equipment use are considered as the main GHG contributing factors. In usage and maintenance processes, vehicles, traffic delays, carbonation, rolling resistance, maintenance treatment, and rehabilitation style are variables that affected the GHG emissions [14,15,16,17,18].

In the field of road engineering, some research frameworks and emission estimation methods have been proposed and used for environmental impact assessment. Globally, there are several tools such as LEEDS and GreenRoad in the US and CEEQUAL and asPECT in the UK that are available to measure the CO_2_ or sustainability. Other tools such as Green Star and DGNB are available in Australia and Germany [11]. 

Yepes presented a study to optimize the cost and CO_2_ emissions of precast/pre-stressed concrete road bridges. The results showed that reducing costs by 1 Euro can save up to 7.5 kg in CO_2_ emissions, and that optimal solutions with lower costs may have a satisfactory environmental outcome and differ only slightly from the optimal solution in terms of environmental impacts [19]. There is a focus on the structure optimization techniques [19,20,21]. Architects and structural engineers try to minimize the embodied energy. Some efforts are being made to reduce the impacts associated with concrete production and consumption [22,23,24,25,26]. Methods for reducing the CO_2_ of concrete are suggested, such as extending the service life of the structure, replacing some of the cement with fly ash or blast furnace slag, and increasing CO_2_ absorption. Some researchers have done work related to CO_2_ absorption from the concrete carbonation during the service life and to the CO_2_ uptake capacity of concrete during recycling. 

While a lot of work has been done worldwide to estimate the GHG emissions related to highway pavement, there is still a need for a method of GHG emission estimation that can be used in cement concrete pavement in China. Moreover, some methods and software are commercial products that are not available for academic research. At present, the main challenge in the study of the environmental impacts of cement concrete pavement in China is the lack of project-validated data and sector-approved methods for life cycle carbon analysis.

## 3. Materials and Methods

### 3.1. Evaluation System Boundary

**Figure 1:**
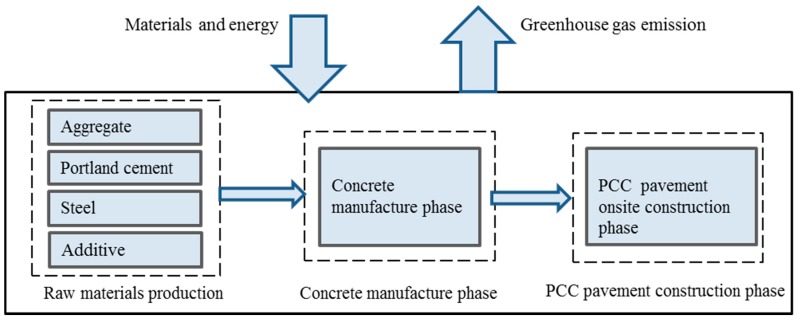
System boundary of PCC pavement construction assessment.

The system boundary of Portland cement concrete (PCC) pavement construction assessment is shown in Figure 1. The research includes the raw materials production, concrete manufacture and PCC pavement onsite construction. This study does not consider the consumption of energy associated with earthwork and the construction of the road base and subbase. 

### 3.2. Definition of GHG

According to the Kyoto Protocol, there are six maim greenhouse gases, namely carbon dioxide (CO_2_), methane (CH_4_), nitrous oxide (N_2_O), hydrofluorocarbons (HFCs), perfluorocarbons (PHCs) and sulphur hexafluoride (SF_6_) [16]. Since HFCs, PFCs and SF_6_ are not commonly present in the emissions from the cement concrete pavement construction process, this study only focuses on three types of GHG: CO_2_, CH_4_ and N_2_O.

### 3.3. Calculation for GHG Emissions

#### 3.3.1. GHG from Limestone Decomposition

In the cement production phase, the limestone decomposition emits CO_2_. The main component of limestone is calcium carbonate (CaCO_3_) and a few other carbonates (such as MgCO_3_). The chemical reaction of decomposition is shown in Equations (1) and (2) to calculate CO_2_ emissions from limestone decomposition.

(1) $$CaCO_{3}=CaO+CO_{2}↑$$

(2) $$MgCO_{3}=MgO+CO_{2}↑$$

The typical CaCO_3_ content in limestone is about 65%, based on chemical Equation (1), and it can be calculated that it will generate 0.44 kg CO_2_ per kg consumption of CaCO_3_. Therefore, in the cement production phase, CO_2_ emissions from decomposition can be calculated as: 65% times 0.44 kg is 0.2860 kg. Likewise, MgCO_3_ content in limestone is about 1.5%, the CO_2_ emission is 7.8 g. Thus, per 1 kg cement production, the CO_2_ emission from limestone decomposition is the sum of 0.286 kg and 7.8 g, which is 0.2938 kg.

#### 3.3.2. GHG Emissions from Energy Consumption

Different types of energy are consumed in the raw materials production, the concrete manufacture phase and the PCC pavement construction phase. The main energy consumed in the process includes coal, fuel and electricity. The GHG emissions are calculated in the Equation (3) [27],

(3) $$E_{GHG}=3.67FQka$$

where E_GHG_ is the GHG emissions in kg; F is the energy consumption in kg, m^3^ or kWh; Q is the embodied energy or calorific value of different energy in MJ/kg, MJ/m^3^ or MJ/kWh; k is the coefficient of carbon emissions; a is the rate of carbon oxygenation. Embodied energy, the coefficient of carbon emissions, and the rate of carbon oxygenation are determined through the relative statistical data from the energy sector in China [28].

Fuel consumption of transportation vehicles can be calculated based on transportation distance and fuel efficiency (different between fully loaded or unloaded situations). For other diesel-powered equipment, fuel consumption can be calculated based on Equation (4). The fuel consumption rate of diesel engines is about 180 g/(hp × h) and 1 hp = 0.735 kW. Based on Equation (4), fuel consumption can be converted into 0.244 kg/kWh:

(4) M=0.244×T×P

where M is the fuel consumption in kg; T is the engine working time in h; P is the engine power in KW.

### 3.4. Global Warming Potential for GHG 

Direct greenhouse gas emissions considered in the study include carbon dioxide (CO_2_), methane (CH_4_) and nitrous oxide (N_2_O). Carbon dioxide is the commonly used measurement unit for global warming or greenhouse gas effects. All other greenhouse gases are referred to as having a “CO_2_ equivalence effect”. This effect has a time horizon due to the atmospheric reactivity or stability of the various contributing gases over time. This study adopted the Intergovernmental Panel on Climate Change’s 100-year time horizon factors as the basis for CO_2_ equivalence for this study. The Global Warming Potential Index (GWPI) values of CO_2_, CH_4_ and N_2_O are 1, 23 and 296, respectively. Therefore, the CH_4_ and N_2_O are converted to the equivalent of CO_2_ following Equation (5).

(5) $$CO_{2}e=E_{GHG}\times \text{GWPI}$$

The total GHG emissions from the cement concrete pavement can be calculated according to Equation (5). 

## 4. Results and Discussions

### 4.1. Case Study 

**Figure 2:**
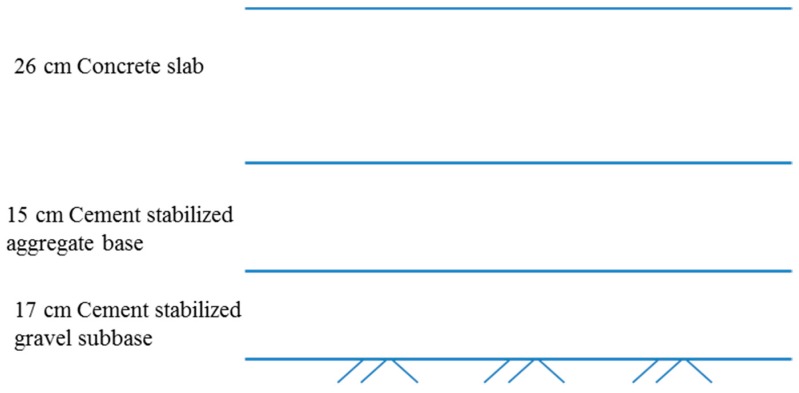
Schematic diagram of the concrete pavement structure.

Using the above inventory analysis results, a case study is carried out on a typical Portland cement concrete pavement of the expressway in the west of China, Shaanxi Province. There are two lanes in each direction, making four lanes in total. The length is 15.2 km, and its width of the subgrade is 28 m. The thickness of the cement concrete pavement layer is 0.26 m. The schematic diagram of the concrete pavement is shown in Figure 2. Additionally, the width of the cement pavement surface is 11.75 m in each direction, which is divided into 4.5 m, 3.75 m, and 3.5 m by the longitudinal joint. The length of each concrete slab is 5.0 m. Transverse shrinkage joints adopt orthorhombic cutting joints which are equipped with steel dowels. Longitudinal shrinkage joints and construction joints are equipped with tie bars. The concrete mix design is a ratio of water to cement of 0.39, the amount of cement is 385 kg/m^3^, the amount of sand is 671 kg/m^3^, the amount of coarse aggregate is 1194 kg/m^3^, the mass ratio of superplasticizers is 0.4%. The transportation distance from the concrete plant to the construction site is an average of 10 km. Ready-mixed concrete is transported by a medium heavy truck. 

### 4.2. GHG Emissions from the Raw Materials Production Phase

#### 4.2.1. Cement

The GHG emissions of cement production are mainly from limestone decomposition, and energy consumption. The GHG of limestone decomposition is calculated following Equations (1) and (2). Different energy types, including coal and electricity, are consumed in the cement production phase. The dry rotary kiln with a production capacity higher than 4000 tons clinker/day is popular in China. The coal is used as fuel for heating the kiln. From the Chinese statistics [28], for one ton of cement, it consumes 116 kg coal and 97.4 KWh electricity. Based on the calorific values, 20,908 KJ/kg for coal and 12,435 KJ/KWh for electricity, it could be calculated that the coal consumption is equal to 2425.33 MJ, and the electricity consumption is equal to 1211.17 MJ. We assume that various raw materials of cement are transported by trucks (5 ton deadweight), and the distance between the quarry and the cement plant is 1 km. Trucks consume about 0.2 L diesel oil in transporting materials for producing one ton of cement which can be converted to 7.165 MJ based on 0.84 kg/L diesel density and 42,652 kJ/kg calorific value. Because the CO_2_ emissions from 1 kg limestone are 0.2938 kg, the CO_2_ emissions from 1235.14 kg limestone consumption are therefore equal to 362.88 kg. According the consumption of coal, diesel and electricity, the GHG emissions inventory of one ton of cement production is listed in Table 1.

From the results of the inventory in Table 1, the CO_2_e emissions from the cement production phase are 1162.50 kg. The CO_2_ emissions from limestone decomposition are about 362.88 kg, accounting for 55.07% of the total CO_2_, and 31.22% of the CO_2_e. 

#### 4.2.2. Aggregate

The GHG emissions in aggregate production are mainly from the electricity production, and the production and combustion of fuel. There are great extents of variance in data, such as the transportation distance and equipment efficiency between different aggregate factories. Additionally, in China, there is a lack of reference data in energy consumption and GHG emissions for aggregate production. The research data from Sjunnesson [29] is used for the calculation. For one ton of aggregate, the GHG emissions value is 5.36 kg CO_2_, which includes 36% emissions from diesel consumption, due to transportation in this phase. The rest of the GHG emissions mainly come from electricity consumption, such as washing, crushing, grinding and the screening machine. Meanwhile, the GHG emissions due to the use of explosives are small. For one ton of coarse aggregate production, it consumes about 0.5 L diesel and 9 KWh electricity. For one ton of sand production, it consumes about 4.59 × 10^−6^ kg coal, 0.025 L diesel and 6.67 × 10^−4^ kWh electricity. The data of GHG emissions of coarse aggregate and sand are shown in Table 2 and Table 3.

#### 4.2.3. Steel

The major production process of steel includes the mining of iron ore, the mineral dressing of iron ore, calcination, steelmaking and rolling manufactures. In addition, this process includes some aided processes such as oxygen generation, coking and other raw material production. Most GHG emissions of steel production are from energy consumption, which includes fuel combustion and energy production. Likewise, coal is a kind of fuel which has higher GHG emissions. Meanwhile, coke and electricity also release GHG (CO_2_, N_2_O and CH_4_). The major sources of GHG include three aspects: the combustion of fossil fuel; fluxes decomposition, such as limestone; and the usage of carbonaceous raw materials, such as electrodes. In this study, the Chinese statistics [28] were used as the reference data to evaluate the GHG emissions from steel production; the details are shown in Table 4. Table 4 only lists some energies. In addition, 2.63 m^3^ natural gas, 808 kg coke, 158 kg heavy oil, and 965 m^3^ coal gas are consumed in one ton of steel production. 

#### 4.2.4. Admixture, Fly Ash and Water

Admixtures are chemical agents which can improve or weaken some properties of cement concrete. There are many additives commonly used for cement concrete, such as water-inducing agents, retardant, early strength agents and air-entraining agents. These additives can improve the workability of cement concrete, cement ductility or strength. These additive agents usually have high emission factors in their production processes. However, the dosage of these agents used in cement concrete is very small. In a typical concrete mix design, the content of additive agents will be no more than 2 L per cubic meter of concrete mix. Though the CO_2_ emission from agents is low, it has been considered in the evaluation. According to European data [30], the energy consumption and GHG emissions inventory for producing 1 kg normal superplasticizers are shown in Table 5. In addition to the coal and diesel and electricity, 91 kg crude oil and 0.21 m^3^ natural gas are consumed in producing 1 kg superplasticizers. 

Fly ash is a commonly used admixture for cement and concrete. Fly ash is a by-product of burning coals, and its components include active silica and alumina. The GHG emissions in the manufacturing of fly ash are mainly derived from the following aspects: direct emissions from fuel consumption of vehicles; and indirect emissions such as the generation of electricity. The specific emissions can be calculated based on related emission factors of fuel and electricity. According to related statistics, the emission of CO_2_ for one ton of fly ash production is 1.51 kg [28], and the main emission source is the consumption of electricity.

Water is an important component in concrete mix design. Though carbon emissions from water production for concrete mix are negligible, water usage is closely associated with cement content; therefore, it indirectly affects the total emissions of the concrete mix production. Water used for concrete mixing can be pumped on site, and it is driven by electricity. Because there is a large difference in water supplying methods, this portion of the GHG emissions is not considered in this study.

### 4.3. GHG Emissions from the Cement Concrete Manufacture Phase

Manufacturing of cement concrete includes mixing and transportation. Mixing equipment is driven by electricity and diesel fuel, and transportation vehicles are powered by diesel. Therefore, energy consumption from concrete production mainly derives from electricity and diesel fuel. For different types of equipment and vehicles, there is a different fuel and electricity consumption rate. Because the cement concrete mixing plant is driven by electricity, GHG emissions mainly derive from electricity production. Based on actual diesel and electricity consumption and their emission factors, GHG emissions from mixing processes can be calculated.

According to a specific project in China, the concrete mix components contain 385 kg/m^3^ cement, 150 kg/m^3^ water, 671 kg/m^3^ sand, 1194 kg/m^3^ coarse aggregate and 0.4% superplasticizers in mass ratio. Assuming that the raw materials’ transportation distance is 10 km, the distance from the mixing plant to the construction site is 10 km as well. The density of diesel is 0.84 kg/L. The electricity consumption of mixing 1 m^3^ concrete is 2 kWh. The total energy consumption and GHG emissions of 1 m^3^ concrete manufacture phase are shown Table 6.

### 4.4. GHG Emissions from the Cement Pavement Onsite Construction Phase

According to technological processes and construction machinery onsite, GHG emissions primarily come from the equipment driven by electricity or diesel. 

The slip-form paver is driven by diesel fuel, and its GHG emissions derive from diesel production and the burning process. When the slip-form paver is working, it requires setting the longitudinal construction joints (dowel steel), the transverse construction joints (tie bar), the expansion joints (sliding dowel steel), the reinforcing steel bar on the edge of the expansion joints, and the corner reinforcing steel bar. Depending on specific construction, rebar processing machinery includes: a steel-bar straightener, a steel cutting machine, a steel bending machine, and a steel welding machine. This processing machinery is driven by electricity, and therefore, the major GHG emissions derive from electricity production. 

Pavement surface finishing adopts a hydraulic roller and mechanical troweling approach. After that, sliding-resistant treatment adopts the concrete pavement texturing machine.

Chinese concrete curing generally adopts straw bags, straws, and jute bags covering the concrete surface. The alternative method is to adopt sand covering on the concrete surface and to spread water to ensure suitable humidity for curing. 

After curing, the concrete layer should be cut by a joint cutter, which is driven by diesel. Filling materials use resin and rubber products. It should be turned into liquid and sealed in the joints. The joint-sealing machine is driven by diesel.

For energy consumption of the equipment and vehicle, the GHG emissions are calculated following Equation (3). Assumptions are made for calculating the total amount of working hours. The width of the cement concrete paver is 9 m in each direction, which is divided into 3 m × 5 m for every board. The thickness of the pavement is 0.25 m. The amount of water for curing is 300 kg/m^3^. The GHG emissions of 1 km of the concrete pavement onsite construction phase are shown in Table 7.

In 1 km of the PCC pavement construction process, the amount of material and energy consumption is calculated according to the specifics of the case study. The results are shown in Table 8. 

**Figure 3:**
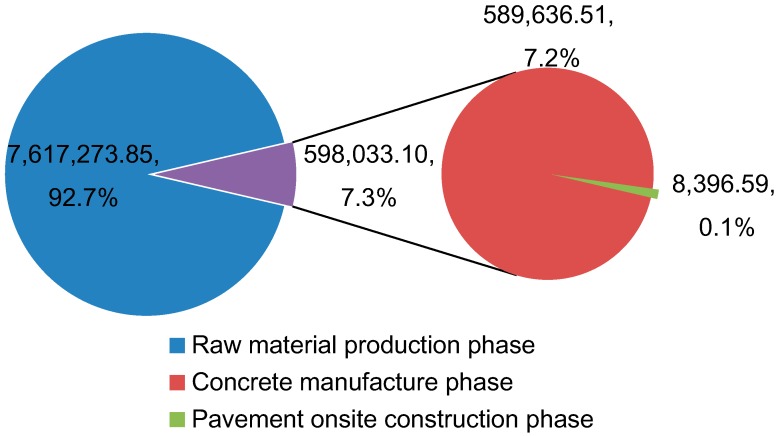
The GHG emissions for the PCC pavement construction process.

In 1 km of the PCC pavement construction process, the GHG emissions are evaluated. The results are shown in Table 9. The total CO_2_e for 1 km of the PCC pavement construction process is 8,215,306.95 kg. The total width of the concrete pavement slab is 23.5 m. For 1-km-long concrete pavement, the surface area is 23,500 m^2^. So the CO_2_e emissions per square meter are around 349.59 kg/m^2^. Additionally, the CO_2_e emissions of the raw material production phase, the concrete manufacture phase and the pavement onsite construction phase are shown in Figure 3.

From the evaluation results, the GHG emissions of the raw material production phase account for 92.7% of the total GHG emissions. The GHG emissions of the concrete manufacture phase account for 7.2%. The 8396.69 kg CO_2_e of the pavement onsite construction phase is only 0.1% of the total GHG emissions. 

### 4.5. Discussions and Recommedations

The results show that 92.7% of the total emissions in the case project came from the raw material production phase, while only 7.3% came from the concrete manufacture phase and the pavement onsite construction phase. The findings are confirmed by the GHG emissions evaluations of highways in previous studies, which indicated that the raw material production accounts for the majority of GHG emissions [18,31,32,33,34]. These data indicated that the focus of carbon reduction in cement concrete pavement should be put on the material production phase. The result is also similar to the GHG emissions from a reinforced concrete-framed building. That research indicated that direct emissions due to onsite construction are relatively small, at only 2.42% of the total GHG emissions, and indirect emissions embedded in the production of building materials, transportation, and offsite human activities are considerably more significant at 97.58% [35]. Therefore, it is a sensible strategy to choose low-carbon building materials. Steel and concrete, as the most popular building and construction materials, contributed to roughly two-thirds of the total GHG emissions. Fly ash, a widely available by-product of coal combustion for electricity generation, can be added to concretes to reduce costs and also to reduce the GWP of concretes [22]. According to Hanson et al. [13], the substitution of fly ash for 25% of the cement in concrete reduced the GWP by 22%. Energy use for cement concrete production (including the transportation and mixing of cement, aggregate, water, and admixtures) is considerably less than the energy for the production of the Portland cement [1]. Different to hot mix asphalt, Portland cement concrete mixes need no drying of the aggregates prior to mixing, as the extra moisture can be accounted for as part of the mix design. Hence, energy for cement concrete production, excluding the production of the raw materials, is mainly for transportation of the raw materials, with a small portion for processing the aggregates at the plant. 

The GHG emissions are related to different approaches for the construction of a secondary concrete road in Greece [36]. The functional unit selected was 1 km of a two-lane urban road with a low traffic load and a total width of 7.3 m. The GHG emissions from the concrete pavement reconstruction process are about 560 t CO_2_e/km. In the research from the US [37], the functional units are based on centerline-kilometers (cl-km). Additionally, a 40 year analysis period is used for the designs. The estimated GWP for rural interstates is 3800 metric ton CO_2_e/cl-km, and the estimated GWP for urban interstates is 6700 metric ton CO_2_e/cl-km. In Stripple’s research [2], the CO_2_ emissions from the construction activities of concrete pavement are around 2.40 E + 09 g/km. There are some differences with the result of 8,215,306.95 CO_2_e/km for PCC pavement construction in this study. The difference is probably derived from different construction methods and technology levels. Additionally, the transportation distances vary evidently. It is noted that pavement construction in different countries is subject to compliance with technical standards, materials availability and practices as usual. The GHG emissions of the PCC pavement construction process may be varied.

## 5. Conclusions

The construction industry and transportation system contribute to a great amount of energy consumption and carbon emissions. The problem in China is that the quantity of the CO_2_ emissions is not clear and the evaluation method is lacking. This study presents a method to calculate the GHG emissions from cement concrete pavement construction. This study proposed an inventory analysis method to evaluate the GHG emissions from Portland cement concrete pavement construction, based on a road project in the west of China. The cement concrete pavement construction was divided into three phases, namely the raw material production phase, the concrete manufacture phase and the pavement onsite construction phase. The GHG emissions of the three phases are analyzed with the life cycle inventory method. The CO_2_e is used to indicate the GHG emissions.

For 1 km of Portland cement concrete pavement construction, the total CO_2_e is 8,215,306.95 kg. Based on the evaluation results, the CO_2_e of the raw material production phase is 7,617,273.85 kg, accounting for 92.7% of the total GHG emissions; the CO_2_e of the concrete manufacture phase is 598,033.10 kg, accounting for 7.2% of the total GHG emissions. Lastly, the CO_2_e of the pavement onsite construction phase is 8396.59 kg, accounting for only 0.1% of the total GHG emissions. The main greenhouse gas is CO_2_ in each phase, which accounts for more than 98% of the total emissions. N_2_O and CH_4_ emissions are relatively insignificant. 

In recent years, studies [20,21,22,23,24,25,26] on reducing CO_2_ emissions have been conducted in different countries in an effort to meet the challenge of global warming. The whole life cycle method is used to evaluate the CO_2_ emissions. In this study, the evaluating boundary is set at the construction stages only. The CO_2_ emissions from pavement in-service and rehabilitation stages are recommended for further work. In the Chinese highway section, the development of a data system related to energy consumption and GHG emissions is still at an early stage. Some methods, such as the replacement of cement by fly ash and blast furnace slag, have been proposed to reduce environmental effects in the cement industry. Cement concrete pavement and cement-treated aggregate base in asphalt pavement are used widely in China, leading to an increase in the energy use and CO_2_ emissions. The development of new energy-efficient and low-carbon technologies and promoting their application in practice will be the key for long-term climate change mitigation strategies. 

## Figures and Tables

**Table 1:** The GHG emissions of one ton of cement production.

Energy Input			GHG Emission			
Coal/kg	Diesel/L	Electricity/KWh	CO_2_/kg	CH_4_/kg	N_2_O/kg	CO_2_e/kg
116	0.20	97.40	659	1.30	1.60	162.50

**Table 2:** The GHG emissions of one ton of coarse aggregate production.

Energy Input		GHG Emission			
Diesel/L	Electricity/kWh	CO_2_/kg	CH_4_/kg	N_2_O/kg	CO_2_e/kg
0.50	9	1.60	1.70	0.014	44.84

**Table 3:** The GHG emissions of one ton of sand production.

Energy Input			GHG Emission			
Coal/kg	Diesel/L	Electricity/kWh	CO_2_/kg	CH_4_/kg	N_2_O/kg	CO_2_e/kg
4.59 × 10^−6^	0.025	6.67 × 10^−4^	0.07	0.38 × 10^−6^	0.6 × 10^−3^	0.25

**Table 4:** The GHG emissions of one ton of steel production.

Energy Input			GHG Emission			
Coal/kg	Diesel/L	Electricity/kWh	CO_2_/kg	CH_4_/kg	N_2_O/kg	CO_2_e/kg	
1466.54	1.56	369.98	3514.96	27.57	37.67	15,299.39	

**Table 5:** The GHG emissions of 1 kg of superplasticizers.

Energy Input		GHG Emission			
Coal/g	Fuel/g	CO_2_/g	CH_4_/g	N_2_O/g	CO_2_e/g
62	74	0.69	1.20	3.50	1064.29

**Table 6:** The GHG emissions of 1 m3 of the concrete manufacture phase.

Energy Input		GHG Emission			
Diesel/L	Electricity/kWh	CO_2_/kg	CH_4_/kg	N_2_O/kg	CO_2_e/kg
12.65	2.00	38.70	0.01	0.12	74.45

**Table 7:** The GHG emissions of 1 km of the concrete pavement onsite construction phase.

Energy Input		GHG Emission			
Diesel/L	Electricity/kWh	CO_2_/kg	CH_4_/kg	N_2_O/kg	CO_2_e/kg
399.73	997.84	2142.15	2.38	8.82	4807.61

**Table 8:** Material and energy consumption for 1 km of the PCC pavement construction process.

Item	Unit	Raw Material Production Phase	Concrete Manufacture Phase	Pavement Onsite Construction Phase
Electricity	kWh	470,297	15,789	247
Coal	kg	368,659	0	0
Diesel	kg	4958	79,895	1203
Water	kg	0	1,184,211	71
Cement	kg	3,039,473	0	0
Sand	kg	5,297,368	0	0
Coarse aggregate	kg	9,426,315	0	0
Concrete	kg	0	18,947,368	0
Steel	kg	241,666	0	0
Superplasticizers	kg	7579	0	0
Energy consumption	GJ	9659	3465	52

**Table 9:** GHG emissions for 1 km of the PCC pavement construction process.

GHG	Unit	Raw Material Production Phase	Concrete Manufacture Phase	Pavement Onsite Construction Phase
CO_2_	kg	3,243,956.24	305,545.18	4322.30
CH_4_	kg	13,914.68	955.29	13.70
N_2_O	kg	11,068.36	57.63	0.83
CO_2_e	kg	7,617,273.85	589,636.51	8396.59
